# Prevention of implant-associated spinal infections: the GAID-protocol

**DOI:** 10.3389/fsurg.2023.1308213

**Published:** 2023-11-23

**Authors:** Joanna Maria Przybyl, Aldemar Andres Hegewald

**Affiliations:** Department of Neurosurgery and Spine Surgery, VAMED Baltic Sea Hospital, Damp, Germany

**Keywords:** surgical site infection, spinal infections, implant-associated infection, sodium hypochlorite/hypochlorous acid, spinal fusion, spondylodiscitis, vertebral osteomyelitis

## Abstract

**Objective:**

The purpose of this study is to investigate the efficacy of the GAID-Protocol, a bundle of intra- and postoperative infection prevention measures, to reduce implant-associated infections in patients undergoing posterior spinal fusion with instrumentation. These preventive measures are organized into a protocol that includes recommendations for four critical areas of implant protection (acronym GAID): Gloves, Antiseptics: sodium hypochlorite/hypochlorous acid (NaOCl/HOCl), Implants and Drainage-use in large wounds.

**Methods:**

We performed a single-site retrospective review of cases undergoing posterior spinal fusion with instrumentation for primarily degenerative spinal diseases before and after implementation of the GAID-Protocol that was specifically designed to protect against implant-associated infections. The primary outcome was postoperative wound complications requiring surgical intervention, with a particular focus on infectious spondylitis/discitis.

**Results:**

230 cases were included: 92 (Group A) before and 138 (Group B) after protocol implementation. Overall, wound complications requiring surgical intervention occurred in 7.6% patients in Group A and in 3.6% patients in Group B (*p* = 0.2297). Of these, infectious spondylitis/discitis was present in 5.4% in Group A and in none of Group B (*p* = 0.0096). The ratio of infectious spondylitis/discitis to other wound problems was 71% to 29% in Group A, while it was 0% to 100% in Group B (*p* = 0.0278). The mean time interval between the first revision surgery for wound complications and hospital discharge was significantly different, 38 days SD 20.3 in Group A and 14.4 days SD 8.6 in Group B (*p* = 0.0442).

**Conclusions:**

In our study, adherence to the GAID-Protocol resulted in a shift from severe to significantly less severe and easier to treat wound complications. Adoption of the GAID-Protocol might contribute to the reduction of implant-associated infections.

## 1. Introduction

Refining measures to prevent surgical site infections is a constant challenge for surgeons. However, evidence-based recommendations for individual preventive measures are difficult to obtain. We generally rely on recommendations from large reviews and meta-analyses, but often, only evidence from methodologically weak studies is available. Moreover, the impact of individual recommendations is often small. To address this problem, so-called “evidence-based care bundles,” i.e., an overall package of meaningful recommendations to reduce surgical site infections, have been proposed and examined with some success in several spinal studies (1–7).

Incidences of surgical site infections are highly dependent on patient risk factors and the type and course of surgical procedures (8, 9). Implant-associated infections of the spine that also affect the vertebral bodies (spondylitis) and/or intervertebral discs (discitis) are associated with complicated treatment courses, high morbidity and with in-hospital mortality rates of up to 15% (10, 11).

We have compiled a bundle of intra- and postoperative infection prevention measures specifically designed to protect against implant-associated infections in often complex and lengthy spine surgeries. These preventive measures are organized into a protocol that includes recommendations for four critical areas of implant protection: Gloves, Antiseptics, Implants and Drainage-use in large wounds—the acronym GAID-Protocol is suggested.

The purpose of this study is to investigate the efficacy of the GAID-Protocol to reduce implant-associated infections in patients undergoing posterior spinal fusion with instrumentation.

## 2. Materials and methods

### 2.1. Study design

We performed a single-site retrospective review of cases undergoing posterior spinal fusion with instrumentation for primarily degenerative spinal diseases from January 2019 to June 2020 (Group A) and after implementation of the new infection prevention measures from July 2020 to October 2022 (Group B). Surgical approaches included conventional open approaches and minimally invasive mini-open approaches. The local ethics committee (Ethikkommissionen bei der Ärztekammer Schleswig-Holstein) was consulted.

A digital search was performed in the hospital information system for all cases that had undergone posterior spinal fusion with instrumentation during the specified periods. Data from the local quality management report was included. The primary outcome was postoperative wound complications requiring surgical intervention, with a particular focus on infectious spondylitis/discitis.

Epifascial wound problems were distinguished from subfascial wound problems, and cases of infectious spondylitis/discitis were considered separately for subfascial findings. The distinction was based on radiologic findings, intraoperative findings, and especially on locally assignable microbiologic findings.

### 2.2. Infection prevention measures

In general, we adhere to the recommendations of the German Commission for Hospital Hygiene and Infection Prevention (12). Prior to July 2020 (Group A), however, the finer points of infection prevention were left to the discretion of the surgeon. From July 2020 onward (Group B), mandatory practices in some areas of intra/postoperative infection prevention have been agreed upon (Table 1). The creation and implementation of the GAID protocol was the result of a quality improvement project in our department. The working group for this project consisted of our hospital hygienist, two experienced operating room nurses and a spine surgeon with an academic background (senior author).

**Table 1:** GAID-Protocol.

Gloves	• Double-gloving.
	• Change outer gloves after sterile draping.
	• Change outer gloves at intervals of 1.5–3 h, before implant manipulation and before wound closure.
	• Avoid touching draped C-arm.
	• Change light handles at intervals of 1.5–3 h or manipulate the light handle with a sterile cloth which is then discarded.
Antiseptics	• Sodium hypochlorite/hypochlorous acid (NaOCl/HOCl):
	- Wound irrigation at intervals of 1.5–3 h and before wound closure. - Disc space irrigation before cage implantation.
	• Iodine-impregnated incision drape.
Implants	• Keep sterile implants and instruments at distance or covered during patient positioning.
	• Implants remain covered until they are inserted.
	• Do not directly touch implants & materials that will remain in the patient. If necessary, use sterile cloth for manipulation.
	• Pre-moisten implants and wires with (NaOCl/HOCl) before insertion.
	• Keep tissues to be re-implanted, such as bone fragments, moist and covered.
Drainage-use in large wounds	• Drainage-use in large wounds from multilevel instrumentations separately in the subfascial and epifascial compartments.
	• Cover drainage outlet points with Iodine-impregnated incision drape.
	• The sub-fascial drainage system should be removed on day 4 at the latest. With minimal drainage volumes and early mobilization of the patient, even earlier.
	• The epi-fascial drainage system is removed when the drainage volume is <30–40 ml/24 h. Regardless of the drainage volume, it is never left in place for more than 10 days.
	• Consider Epidermal VAC (Vacuum Assisted Closure) Dressings in High Risk Patients.

Table 1 shows the new infection prevention measures for instrumented spinal surgeries agreed from July 2020. The acronym GAID is suggested.

### 2.3. Statistical analysis

Statistical analyses were performed using Prism 7.0e for Mac OS X, GraphPad Software, La Jolla California USA. Descriptive statistics including mean, median, minimum, maximum, standard deviation and interquartile range were calculated. For statistical testing between groups for categorical variables Fisher’s exact tests or chi-square tests were performed, for continuous variables Mann–Whitney tests or *t*-tests for unpaired samples were performed. All statistical tests were two-sided, and a *p*-value < 0.05 was considered statistically significant. Figures were created with the same software version.

## 3. Results

230 patients with posterior spinal fusion with instrumentation for primarily degenerative spinal diseases were included in this retrospective study. 92 surgeries were performed before (Group A) and 138 were performed after (Group B) implementation of the new infection prevention measures. The mean documented follow-up time at our institution was 22 months SD 17 (Group A) and 12 months SD 8 (Group B).

No significant differences were found between the groups with regard to demographic data (age, sex) and risk factors (body mass index, diabetes, rheumatologic diseases, smoking, previous spine surgery) (Table 2). Both groups presented with severe disability according to ODI. There were no differences in the main indications for spine surgery, with the exception of recurrent disc herniation (*p* = 0.0215).

**Table 2:** Patient data.

	Group A	Group B	*P* value
Number of patients	92	138	
Demographics			
Age at surgery (years; mean SD; range)	60 (15; 26–82)	62 (13; 27–82)	0.2638^a^
Sex			>0.9999^b^
Male	46%	46%	
Female	54%	54%	
Risk factors			
BMI (mean SD; range)	28 (4; 19–40)	30 (5; 18–43)	0.0814^a^
Obesity (BMI ≥ 30)	33%	45%	0.1171^b^
Diabetes	14%	12%	0.6857^b^
Rheumatological disease	10%	9%	0.8177^b^
Smoking	29%	23%	0.3553^b^
Previous spine surgeries	40%	36%	0.4893^b^
Preoperative oswestry disability index (mean SD; range)	45 (19; 8–98)	47 (16; 8–89)	0.2993^a^
Main indications for instrumented spinal surgery			
Foraminal stenosis (often associated with spondylolisthesis or segmental scoliotic deformity)	28%	30%	0.8826^b^
Degenerative deformity	13%	16%	0.5758^b^
Degenerative pathological hypermobility	11%	12%	>0.9999^b^
Spondylolisthesis	10%	6%	0.3069^b^
Central canal stenosis (often associated with myelopathy)	9%	7%	0.6102^b^
Recurrent disc herniations	11%	3%	**0**.**0215**^b^
Modic endplate changes	5%	7%	0.7870^b^
Adjacent segment pathology	3%	9%	0.1103^b^
Implant failure	5%	7%	0.7870^b^
Spinal fracture	1%	4%	0.4060^b^
Primary spondylodiscitis	1%	0%	0.4000^b^
Postoperative spondylodiscitis	0%	1%	>0.9999^b^
Reinstrumentation after implant-associated infection	2%	1%	0.5655^b^

Table 2 illustrates patient data, major preoperative risk factors and pathological findings triggering our decision for instrumented spinal surgery. BMI, body mass index; SD, standard deviation.

*P* values in bold are considered statistically significant test results (*p* < 0.05).

^a^ *t* test.

^b^ Fisher’s exact test.

### 3.1. Surgical data

In general, we found comparable results in surgical data (Table 3). However, modest differences were found in the number of dorsally instrumented levels, ranging from 1 to 15 levels, with a mean of 2.1 SD 1.9 in Group A and 2.4 SD 2.0 in Group B (*p* = 0.0241), as well as in surgical time (*p* = 0.0139) and duration of wound drainage (*p* < 0.0001).

**Table 3:** Surgical data.

	Group A	Group B	*P* value
Number of patients	92	138	
Dorsally instrumented levels (mean SD; range)	2.1 (1.9; 1–9)	2.4 (2.0; 1–15)	**0**.**0241**^b^
1–2 levels	78%	67%	0.1587^c^
3–4 levels	14%	25%	
5–8 levels	4%	7%	
>8 levels	3%	2%	
Intervertebral cages (mean SD; range)	0.8 (0.7; 0–3)	0.9 (0.7; 0–4)	0.1270^b^
Dorsal mini-open approaches	24%	35%	0.0820^a^
Lumbar levels	91%	91%	>0.9999^a^
Thoracic levels	10%	11%	0.8299^a^
Cervical levels	3%	6%	0.5325^a^
Surgical time (minutes; mean SD; range)	286 (94; 137–556)	312 (95; 99–797)	**0**.**0139**^b^
Estimated blood loss^d^			
<100 ml	32%	38%	0.6302^c^
100–500 ml	39%	33%	
500–1,000 ml	20%	24%	
>1,000 ml	9%	5%	
Wound drainage	47%	56%	0.1817^a^
Wound drainage (days; mean SD; range)^e^	2.8 (1.3; 1–6)	4.2 (1.3; 2–7)	**<0**.**0001**^b^

Table 3 reports surgical data.

SD, standard deviation.

*P* values in bold are considered statistically significant test results (*p* < 0.05).

^a^ Fisher’s exact test.

^b^ Mann–Whitney test.

^c^ Chi-square test.

^d^ Incomplete data 53% (44% Group A, 59% Group B).

^e^ Incomplete data: 52% (47% Group A, 56% Group B).

### 3.2. Surgical site infections

**Figure 1:**
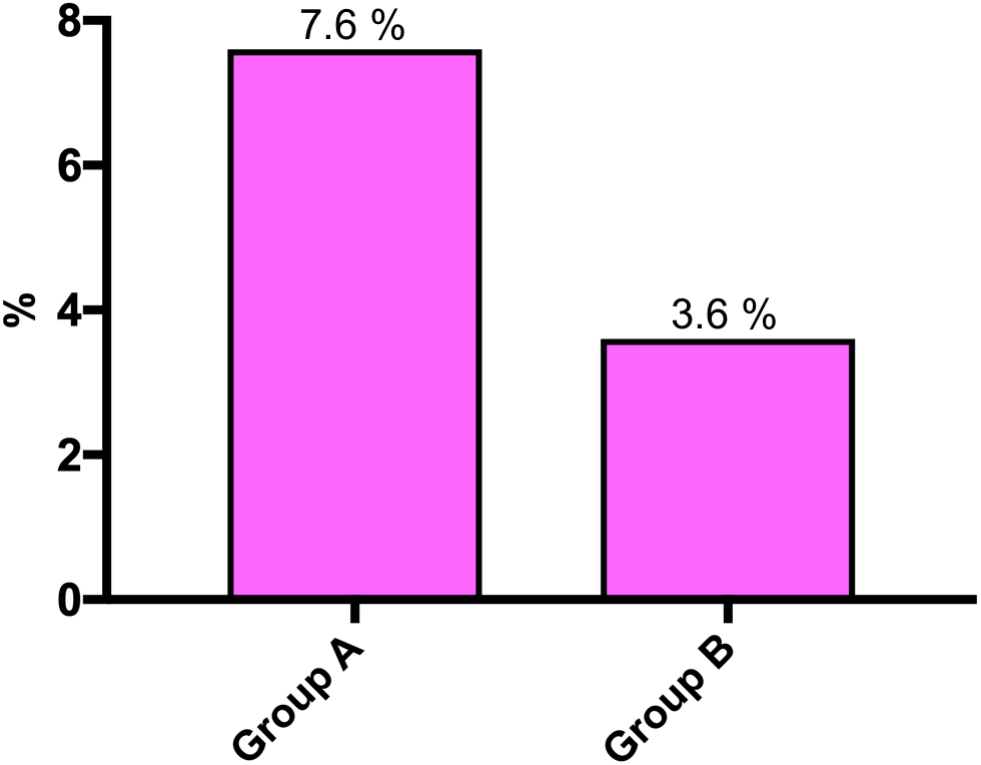
Wound complications requiring surgical intervention before (Group A) and after (Group B) implementation of the GAID protocol (*p* = 0.2297).

Overall, wound complications requiring surgical intervention occurred in 7 (7.6%) patients in Group A and in 5 (3.6%) patients in Group B (*p* = 0.2297) (Figure 1).

**Figure 2:**
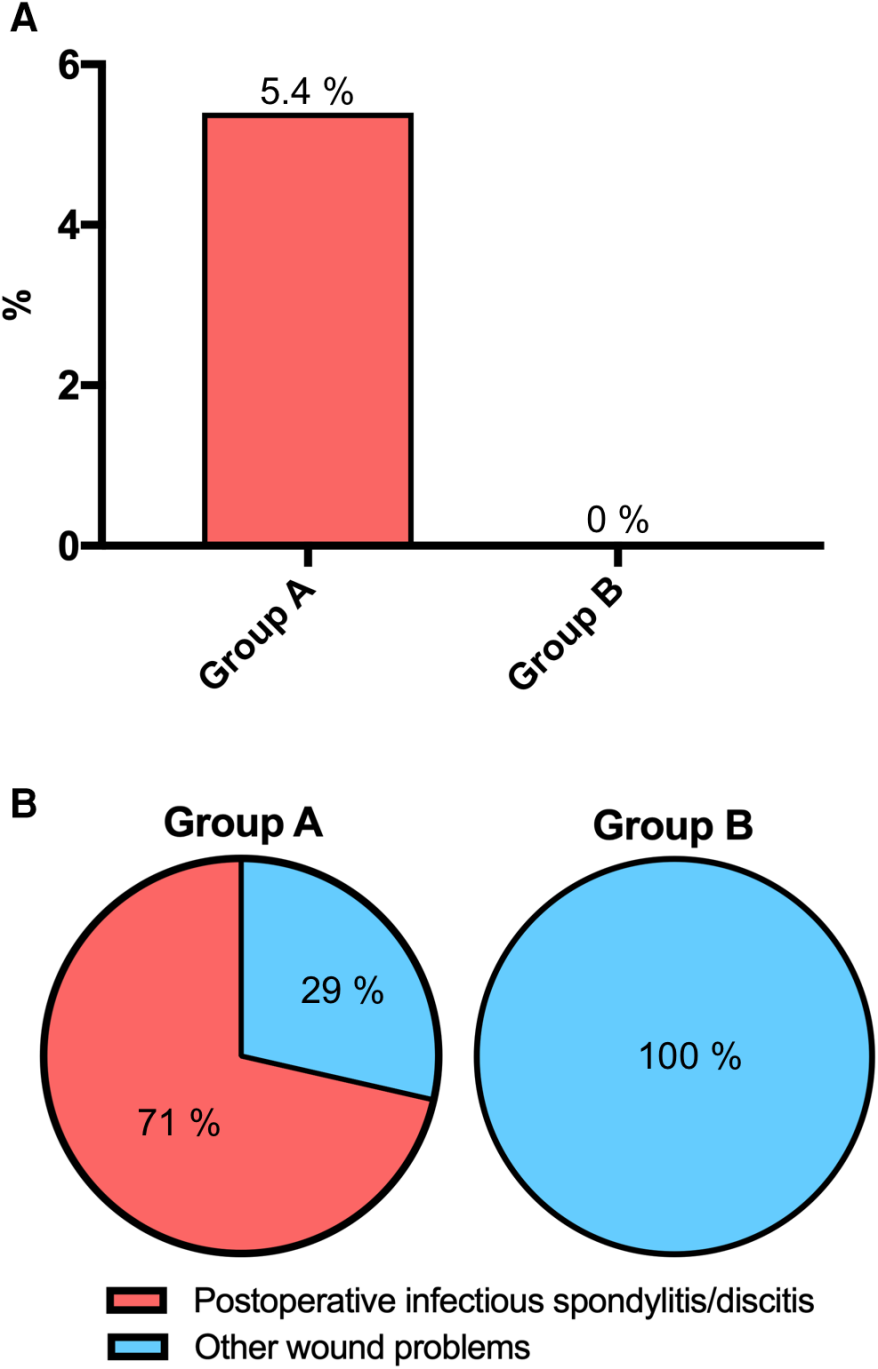
Postoperative infectious spondylitis/discitis before (Group A) and after (Group B) implementation of the GAID protocol (*p* = 0.0096) (**A**), and ratios of infectious spondylitis/discitis to other wound problems (*p* = 0.0278) (**B**).

Of these, infectious spondylitis/discitis was present in 5 (5.4%) in Group A and in none of Group B (*p* = 0.0096) (Figure 2A).

The ratio of infectious spondylitis/discitis to other wound problems was 5 (71%) to 2 (29%) in Group A, while it was 0% to 5% (100%) in Group B (*p* = 0.0278) (Figure 2B).

With regard to demographic data, risk factors and surgical data, patients with wound complications showed comparable results in Groups A and B (Table 4). The median time interval between index surgery to revision surgery for wound complications was 20 days IQR 8–140 for Group A and 21 days IQR 13–42 for Group B (*p* = 0.9596).

**Table 4:** Patients with surgical site infections.

	Group A	Group B	*P* value
Number of wound complications	7	5	
Patient data			
Age at surgery (years; mean SD; range)	61 (11; 47–79)	68 (7; 59–75)	0.2590^a^
Sex			0.5758^b^
Male	43%	20%	
Female	57%	80%	
BMI (mean SD; range)	30 (5; 26–39)	31 (5; 26–38)	0.7944^a^
Obesity (BMI ≥ 30)	43%	60%	>0.9999^b^
Diabetes	14%	40%	0.5227^b^
Rheumatological disease	29%	40%	>0.9999^b^
Smoking	14%	0%	>0.9999^b^
Previous spine surgeries	29%	40%	>0.9999^b^
Surgical data			
Dorsally instrumented levels (mean SD; range)	2 (1.2; 1–4)	2.4 (2.0; 1–5)	0.9621^c^
Surgical time (minutes; mean SD; range)	303 (71; 216–420)	319 (71; 223–380)	0.5303^c^
Wound drainage	43%	40%	>0.9999^b^
Time intervals			
Time index surgery to revision surgery (days; median IQR; range)	20 (8–140; 8–219)	21 (13–42; 9–48)	0.9596^c^
Time Revision Surgery To Hospital Discharge (Days; Mean SD; Range)	38.0 (20.3; 8–66)	14.4 (8.6; 1–24)	**0**.**0442**^c^

Table 4 illustrates characteristics of patients with surgical site infections. SD, standard deviation; BMI, body mass index; IQR, interquartile range.

*P* values in bold are considered statistically significant test results (*p* < 0.05).

^a^ *t* Test.

^b^ Fisher's Exact test.

^c^ Mann–Whitney test.

^d^ Chi-square Test.

**Figure 3:**
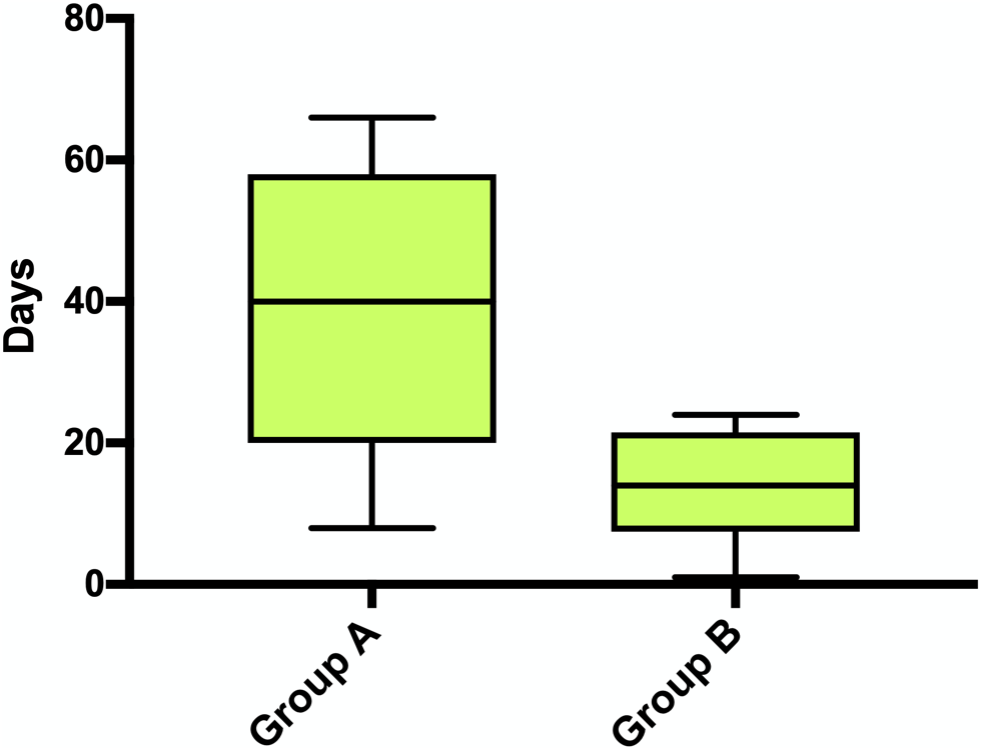
Time interval between the first revision surgery for wound complications and hospital discharge (*p* = 0.0442).

The mean time interval between the first revision surgery for wound complications and hospital discharge was significantly different, 38 days SD 20.3 in Group A and 14.4 days SD 8.6 in Group B (*P* = 0.0442) (Figure 3, Table 4). In-hospital mortality rate was 0% in both groups.

Descriptive microbiological data is provided in (Table 5).

**Table 5:** Microbiological data.

	Group A	Group B
Number of wound complications	7	5
Patients		
1	Scattered Staph. epidermidis (multiresistent) in all samples, subfascial.	Scattered Streptococcus dysgalaktiae in 2 of 9 samples, epifascial.
2	After enrichment Cutibacterium acnes in all samples, subfascial & on screws.	Scattered Staph. epidermidis in 1 of 4 samples, subfascial.
3	No bacteria detected, epifascial.	Scattered Klebsiella oxytoca in all samples, epifascial.
4	Scattered Peptostreptococcus harei & Staph. epidermidis (multiresistent) in all samples, subfascial & on screws.	After enrichment Staph. aureus in all samples, subfascial.
5	Moderate Cutibacterium acnes, Staph. epidermidis, Staph. simulans, Staph. capitis & Staph. hominis in all samples, subfascial, on cage & on screws.	Abundant Staph. aureus in 1 of 3 samples, epifascial.
6	Abundant E. coli in all samples, subfascial, disc & in screwholes.	
7	After Enrichment Cutibacterium Acnes & Staph. Capitis In 7 Of 11 Samples, Subfascial, On Cage & On Screws.	

Table 5 shows descriptive microbiological data.

No adverse effects of the antiseptic sodium hypochlorite/hypochlorous acid or of the iodine-impregnated incision drape were observed.

## 4. Discussion

We investigated intra- and postoperative infection prevention measures (GAID-Protocol) specifically designed to protect against implant-associated infections. The study population consisted of elderly patients with severe disabilities according to the Oswestry Disability Index (46 SD 17) with a high rate of previously performed spine surgeries (37%) who underwent posterior spinal fusion with instrumentation for mainly degenerative spinal disorders. 92 of the surgeries (Group A) were performed before and 138 of the surgeries (Group B) were performed after the new infection prevention measures were implemented. Groups A and B had comparable characteristics in terms of patient data and surgical data (Tables 2, 3) and showed high risk factors for surgical site infections in terms of prevalence of known risk factors such as obesity (33%/45%), diabetes (14%/12%), rheumatological disease (10%/9%), and smoking (29%/23%), respectively (8, 13, 14).

In our study, a significant decrease in severe surgical site infections related to infectious spondylitis/discitis from 5.4% (Group A) to zero (Group B) was observed (*p* = 0.0096), (Figure 2A).

Wound complications requiring surgical intervention were observed in 7.6% (Group A) and 3.6% (Group B), with no statistically significant difference (*p* = 0.2297). However, the ratio of infectious spondylitis/discitis to other wound problems, most of which were much less severe, was 71% to 29% in Group A, whereas it was 0% to 100% in Group B (*p* = 0.0278), (Figure 2B). Thus, adherence to the GAID-Protocol resulted in a shift from severe to significantly less severe and easier to treat wound complications. This is also indicated by the lower mean time interval between first revision surgery and hospital discharge of 14.4 days SD 8.6 in Group B compared with 38 days SD 20.3 in Group A (*p* = 0.0442), (Figure 3, Table 4). Because of the small sample size, meaningful comparison of descriptive microbiologic data is difficult, but the trends toward more epifascial wound complications and fewer bacteria-positive samples in Group B may underscore our observations (Table 5).

### 4.1. Care bundles to prevent surgical site infections

The introduction of car bundles, i.e., an overall package of meaningful recommendations for action, has led to significant reductions in surgical site infection rates in several studies (Table 6) (1–7).

**Table 6:** Studies reporting implementation of care bundles.

Studies (references)	Care bundles	SSI rate without/with care bundle	Surgical procedures
(1)	Preoperative nasal mupirocin and chlorohexidine body decontaminations, storage optimization of operating room supplies, preoperative antibiotic administration algorithm, staff training on povidone-iodine scrub and paint, intrawound vancomycin in instrumented cases, postoperative early patient mobilization, wound checks at 2 and 6 weeks postoperatively.	4.1%/2% *p = 0.01*	Discectomy, decompression, spinal fusion, vertebral augmentation.
(2)	Only in high risk patients (19%): preoperative nasal mupirocin and chlorohexidine body decontaminations, additional intravenous vancomycin prophylaxis, diluted povidone-iodine wound irrigation.	3.8%/0.7% *p < 0.01*	Spinal instrumentation surgeries.
(3)	Sterile technique for surgical dressing changes, dressings to be changed daily for 7 days, physician awareness program/surveillance feedback.	3.8%/2.1% *p = 0.03*	Spinal fusion.
(4)	5–7 days hydrocellular foam dressing, early mobilization, staff training, use of surveillance feedback.	19.4%/2.6% *p = 0.001*	Lumbar surgery: disc herniation, spinal stenosis, spondylolisthesis, scoliosis, kyphosis, trauma.
(5)	Preoperative: chlorhexidine scrubs, screening with nasal swabbing, and decolonization of S. aureus. Perioperative antibiotic administration. Diluted povidone-iodine wound irrigation, application of intrawound vancomycin powder.	6%/<2% *p not reported*	Laminectomies, spinal fusions.
(6)	Best Practice Guideline (BPG) for surgical site infection (SSI) prevention in high-risk pediatric spine surgery (15).	8%/1% *p = 0.005*	Spinal fusion for neuromuscular scoliosis.
(7)	Best Practice Guideline (BPG) for surgical site infection (SSI) prevention in high-risk pediatric spine surgery (15).	16.1%/4.4% *p = 0.005*	Spinal fusion for neuromuscular scoliosis.

Table 6 lists studies reporting the implementation of care bundles to prevent surgical site infections (SSI) in spinal surgery.

A number of these studies do not classify surgical site infections into subgroups (1, 3, 5). A common classification is based on the Centers for Disease Control and Prevention (United States of America) definitions of superficial, deep and organ/space infections, which can be roughly translated into epifascial, subfascial and vertebral bone/disc infections in the spine (16).

Two of these studies reported similar findings to ours in terms of a shift from severe to less severe wound complications (2, 6). Glotzbecker et al. reported the implementation of a best practice guideline (15) for the prevention of surgical site infections in spinal fusion for neuromuscular scoliosis. The overall infection rate did not change significantly from 9% to 6%, but there was a significant shift from deep to superficial infections before and after implementation of the best practice guideline. The ratio of deep to superficial infections changed from 92% to 8% before implementation to 14%–86% after implementation (6). Similarly, Yamada et al. reported on the implementation of a preventive care bundle in spinal instrumentation surgery. The ratio of deep site and/or organ infections to superficial infections changed from 75% to 25% before implementation to 50%–50% after implementation (2). Similar to our study, both investigators used an antiseptic, in their cases diluted povidone-iodine, for wound irrigation as part of their care bundle.

There are a number of problems in interpreting and comparing the results of studies in this area, as there is a great deal of heterogeneity in terms of patient characteristics, surgical procedures, and agreement in the field on what basic infection prevention measures are, and which are being studied in terms of new adaptation. This leads to confounding factors that are difficult to control in these mostly retrospective observational studies. Table 6 also shows the wide variety of infection prevention measures that have been developed for the preoperative, intraoperative and postoperative settings. Each individual prevention measure can play a critical role in orchestrating an effective strategy to reduce surgical site infections. Investigating each prevention intervention separately in large randomized controlled trials, however, is difficult to realize in practice and could be ethically questionable in some cases. In this light, pragmatic testing of carefully selected care bundles for their ability to reduce surgical site infections makes sense and could be an important future strategy.

### 4.2. GAID-protocol

Previous care bundles for the prevention of wound infections contain general recommendations such as preoperative nasal and body decontamination and structured postoperative wound care (Table 6). These are part of our basic prevention measures, for which we generally follow the recommendations of the German Commission for Hospital Hygiene and Infection Prevention (12). Another recommended source is the Global Guidelines for Surgical Site Infections of the World Health Organization (17). More specific recommendations include the use of antiseptics or intrawound vancomycin powder (1, 2, 5–7). Interestingly, staff training and surveillance feedback appear to play an important role in reducing surgical site infections (3, 4).

As an important complement to our basic prevention measures, a bundle of intra- and postoperative infection prevention measures has been defined specifically to protect against implant-associated infections in spine surgery. These preventive measures are organized in a protocol that includes recommendations for four critical areas of implant protection: gloves, antiseptics, implants and drainage-use in large wounds (Table 1).

#### 4.2.1. Gloves

Surgical gloves can be perforated unnoticed or noticed in up to 40% of surgical procedures (18). Therefore, we advocate the wearing of two gloves (double-gloving) for surgeons and surgical nurses. After surgical access alone, bacterial contamination was found in more than 12% of gloves in some studies of orthopedic prosthesis implantation (19, 20). In an observational study of 389 lumbar fusions, changing outer gloves during double gloving prior to implant placement demonstrated a significant reduction in wound infections (21). Two reviews focusing on spine surgery recommend double gloving with 2 h glove changes and changes before contact with implants (22, 23). Other sources of glove contamination include light handles and C-arm covers. Contamination in 15%–50% of sterile light handles has been noted in orthopedic procedures (24, 25). Contamination of the sterile C-arm drape is to be expected already during draping and as the duration of the procedure increases (26). Contact of the sterile C-arm drapes with gloves, instruments, or the surgical field should be avoided. The wound area should always be covered during radiographs and 3D rotations.

#### 4.2.2. Antiseptics

Decontamination of the surgical wound is attempted with either antiseptic irrigation or intrawound antibiotics.

The results of a systematic review indicated that topical vancomycin application may be a potential strategy to reduce the incidence of surgical site infections in spine surgery. However, its use is mainly based on retrospective studies with some methodological weaknesses that do not allow for firm conclusions (27). Experimental studies suggest cytotoxic effects of topical application of vancomycin (28). However, the major concern of widespread use is the emergence of vancomycin-resistant organisms and pressure for more gram-negative and polymicrobial infections at the surgical site. A meta-analysis found that topical vancomycin powder may reduce the overall rate of wound infections from 3.8% to 2.3% (OR 0.60; 95% CI 0.51–0.71; *p* < 0.05): but with the accompanying reduction in the rate of Gram-positive wound infections, the risk for developing more difficult-to-treat Gram-negative or polymicrobial wound infections was nearly twice as high in the topical vancomycin group (29). Therefore, the authors recommend limiting its use to patients who need it most because of high risk factors.

A significant reduction in wound infection rates can be achieved with different antiseptic irrigations prior to wound closure (12). Three other reviews conclude that the available literature shows that the use of intraoperative topical antiseptics is of clinical relevance to prevent infection of orthopedic implants (30–32). A meta-analysis of 20 studies of instrumented spine surgery showed that topical antibiotics can reduce the risk by threefold, while antiseptic irrigation with povidone-iodine can reduce the risk of wound infection by sevenfold (33). For example, an observational study of 323 spine surgeries showed a significant reduction in deep wound infections after establishing 90 s wound irrigation every 1.5 h using 1% povidone-iodine (34). In addition, povidone-iodine showed comparable to better results than vancomycin in preventing postoperative infections in a network meta-analysis (32). In conclusion, antiseptics are, in our view, the better choice for topical infection prophylaxis.

##### 4.2.2.1. Sodium hypochlorite/hypochlorous acid (NaOCl/HOCl)

In our clinical work, we chose 0.08% NaOCl/HOCl, a newer antiseptic, in which we saw potential advantages compared with commonly used antiseptics. Kramer et al. provide an excellent review and consensus recommendation with characterization and comparison of antiseptics suitable for intraoperative use (35). There, NaOCl/HOCl is reported as highly effective against vegetative bacteria, bacterial spores, aspergilli, oocysts of cryptosporidium, and enveloped viruses. In efficacy against biofilms, NaOCl/HOCl is described to be more effective than a polyhexanide/betaine combination. Onset of action occurs more rapidly than with povidone-iodine, octenidine, and polyhexanide (35) in between 30 s and 5 min (36). Unlike commonly used surface-active antiseptics, NaOCl/HOCl acts by a physiological bactericidal mechanism, leaving only NaCl and water as end products (37). Better biocompatibility and less cytotoxicity as well as improved wound healing compared to povidone-iodine have been reported in experimental and clinical studies (35), but literature for spine surgery applications is lacking. Unlike established antiseptics such as povidone iodine or polyhexanide, which are contraindicated for use on nervous tissue, NaOCl/HOCl is considered compatible for this application (35). In summary, NaOCl/HOCl appears to us to be a modern antiseptic with the best properties for use in spinal surgery.

##### 4.2.2.2. Intervertebral disc as a possible source of infection

An analysis of 169 discs from 87 patients operated on for non-inflammatory spinal problems showed bacteria-positive cultures in 45% (20% P. acnes, 18% coagulase-negative staphylococci, 7% other). Patients who underwent surgery for disc herniation and degenerative disc disease showed a significant association with positive bacterial cultures compared to control patients (trauma, deformities) (*x*^2^ = 15.37; *p* = 0.000088) (38). In addition, a similar study of 368 patients undergoing disc herniation surgery demonstrated the formation of a biofilm in the disc by P. acnes (39). Because intravenous antibiotic prophylaxis does not reliably achieve sufficient bactericidal doses in the disc space (40, 41), topical application of effective antiseptics (42) to the disc space prior to implant placement appears reasonable, although not yet proven. Therefore, we flush the disc space with NaOCl/HOCl during nucleotomy and disc preparation and before cage implantation.

##### 4.2.2.3. Iodine-impregnated incision drape

The use of non-antiseptically impregnated incision drape significantly increases the risk of wound infection, which is why this application is not recommended (12). However, iodine-impregnated incision drapes are antimicrobial, with iodine penetrating into deeper skin layers (43). Recent randomized trials confirmed a significant reduction in wound contamination (44, 45).

#### 4.2.3. Implants

Pedicle screws placed on the instrument table showed initial contamination with staphylococci and micrococci after only 20 min (46). Similarly, other studies showed contamination rates of up to 55% in exposed implants, which could be significantly reduced by simply covering them (23, 47–49). To protect implants and sterile instruments, it should be considered that patient positioning can result in more than 4-fold higher bacterial concentrations in the air in the operating room and in some cases exceed the standards for ultra-clean air (50).

#### 4.2.4. Drainage-use in large wounds

In our clinical practice, we usually use drainages only in large wounds with conventional open spinal instrumentations. We adapted a drainage management protocol of Ward et al. who reported a reduction in wound complication rates from 19% to 0% in a study of 76 cases of neuromuscular scoliosis surgery (51), (Table 1). It should be noted here, that some studies have indeed shown a correlation of the incidence of wound infection with the duration of drainage placement (52, 53). High-quality reviews, however, show that there is no consistent, let alone high-quality, evidence on the impact of longer position times of wound drainages on wound infection rates after spine surgery (54). The varying results in this question may result from the dressing technique at the drainage exit site. Commonly used, simple bandage dressings are quickly perfused or peel off in bed. This quickly creates an entry point for germs. Therefore, we additionally cover the drainage dressing with an iodine-impregnated incision drape, which can remain in place for up to 5 days. Dressing changes should be performed using aseptic technique. There is no evidence-based literature for this approach yet. We have not observed any adverse effects with this procedure and it seems to be well tolerated by our patients.

Epidermal VAC (Vacuum Assisted Closure) dressings for spinal fusion reduced wound dehiscence and infection in first observational studies (55, 56), especially in cases at high risk for infection (57). We agree that primarily, epidermal VAC therapy is appropriate for patients who are at high risk for infection and/or wound dehiscence (58).

### 4.3. Limitations and strength of this study

Our study compares two well-characterized cohorts of patients who are well matched for patient and surgical data. Our organizational structure provided high compliance with the GAID-Protocol. Limitations include those inherent to a retrospective report using historical controls. These include a general increased awareness of the medical team after implementation of the GAID-Protocol as a confounding factor. The small overall sample size provides only limited statistical power. We acknowledge that our data set is not able to clearly distinguish the additive effect of each measure, but we believe that the cumulative effect is compelling. Moreover, each of these measures is low-risk and low-cost, so we do not believe there are any relevant drawbacks to adopting the GAID protocol. Further higher-powered studies would be useful to further evaluate the GAID-Protocol or components of it, such as the use of NaOCl/HOCl for wound and disc space irrigation.

## 5. Conclusions

In our study, adherence to the GAID-Protocol, a bundle of infection prevention measures specifically designed to protect against implant-associated infections in spine surgery, resulted in a shift from severe to significantly less severe and easier to treat wound complications. A significant decrease in severe surgical site infections related to infectious spondylitis/discitis from 5.4% to zero was observed. Adoption of the GAID-Protocol might contribute to the reduction of implant-associated infections.

## Data Availability

The ananomized raw data supporting the conclusions of this article will be made available by the authors, with undue reservation.

## References

[1] Featherall J Miller JA Bennett EE Lubelski D Wang H Khalaf T Implementation of an infection prevention bundle to reduce surgical site infections and cost following spine surgery. JAMA Surg. (2016) 151:988–90. 10.1001/jamasurg.2016.1794 27438940

[2] Yamada K Abe H Higashikawa A Tonosu J Kuniya T Nakajima K Evidence-based care bundles for preventing surgical site infections in spinal instrumentation surgery. Spine (Phila Pa 1976). (2018) 43:1765–73. 10.1097/BRS.0000000000002709 29794586

[3] Agarwal N Agarwal P Querry A Mazurkiewicz A Tempel ZJ Friedlander RM Implementation of an infection prevention bundle and increased physician awareness improves surgical outcomes and reduces costs associated with spine surgery. J Neurosurg Spine. (2018) 29:108–14. 10.3171/2017.11.SPINE17436 29701563

[4] Castellà L Sopena N Rodriguez-Montserrat D Alonso-Fernández S Cavanilles JM Iborra M Intervention to reduce the incidence of surgical site infection in spine surgery. Am J Infect Control. (2019) 48(5):550–4. 10.1016/j.ajic.2019.09.007 31706545

[5] Tomov M Wanderman N Berbari E Currier B Yaszemski M Nassr A An empiric analysis of 5 counter measures against surgical site infections following spine surgery-a pragmatic approach and review of the literature. Spine J. (2019) 19:267–75. 10.1016/j.spinee.2018.05.043 29864545

[6] Glotzbecker M Troy M Miller P Berry J Cohen L Gryzwna A Implementing a multidisciplinary clinical pathway can reduce the deep surgical site infection rate after posterior spinal fusion in high-risk patients. Spine Deform. (2019) 7:33–9. 10.1016/j.jspd.2018.06.010 30587318

[7] Stephan SR Illingworth KD Gupta K Andras LM Skaggs DL. Surgical site infection following neuromuscular posterior spinal fusion fell 72% after adopting the 2013 best practice guidelines. Spine (Phila Pa 1976). (2021) 46:1147–53. 10.1097/BRS.0000000000004050 33826592

[8] Yao R Zhou H Choma TJ Kwon BK Street J. Surgical site infection in spine surgery: who is at risk. Global Spine J. (2018) 8:5S–30S. 10.1177/2192568218799056 30574441 PMC6295819

[9] Zhou J Wang R Huo X Xiong W Kang L Xue Y. Incidence of surgical site infection after spine surgery: a systematic review and meta-analysis. Spine (Phila Pa 1976). (2020) 45:208–16. 10.1097/BRS.0000000000003218 31464972

[10] Lang S Frömming A Walter N Freigang V Neumann C Loibl M Is there a difference in clinical features, microbiological epidemiology and effective empiric antimicrobial therapy comparing healthcare-associated and community-acquired vertebral osteomyelitis. Antibiotics (Basel). (2021) 10:1410. 10.3390/antibiotics10111410 34827348 PMC8615006

[11] Lang S Walter N Schindler M Baertl S Szymski D Loibl M The epidemiology of spondylodiscitis in Germany: a descriptive report of incidence rates, pathogens, in-hospital mortality, and hospital stays between 2010 and 2020. J Clin Med. (2023) 12:3373. 10.3390/jcm12103373 37240479 PMC10219516

[12] Kommission für Krankenhaushygiene und Infektionsprävention (KRINKO) beim Robert Koch-Institut. Prävention postoperativer wundinfektionen. Bundesgesundheitsblatt—Gesundheitsforschung—Gesundheitsschutz. (2018) 61:448–73. 10.1007/s00103-018-2706-2 29589090

[13] Koyama K Ohba T Ebata S Haro H. Postoperative surgical infection after spinal surgery in rheumatoid arthritis. Orthopedics. (2016) 39:e430–3. 10.3928/01477447-20160404-05 27064779

[14] Echt M De la Garza Ramos R Nakhla J Gelfand Y Cezayirli P Holland R The effect of cigarette smoking on wound complications after single-level posterolateral and interbody fusion for spondylolisthesis. World Neurosurg. (2018) 116:e824–9. 10.1016/j.wneu.2018.05.103 29803058

[15] Vitale MG Riedel MD Glotzbecker MP Matsumoto H Roye DP Akbarnia BA Building consensus: development of a best practice guideline (BPG) for surgical site infection (SSI) prevention in high-risk pediatric spine surgery. J Pediatr Orthop. (2013) 33:471–8. 10.1097/BPO.0b013e3182840de2 23752142

[16] National Healthcare Safety Network. *Surgical Site Infection Event (SSI)*. (2023). Available at: http://www.cdc.gov/nhsn/pdfs/pscmanual/9pscssicurrent.pdf

[17] World Health Organization. Global guidelines for the prevention of surgical site infection. 2nd ed. World Health Organization (2018). Available at: https://www.who.int/publications/i/item/9789241550475 30689333

[18] Kommission für Krankenhaushygiene und Infektionsprävention (KRINKO) beim Robert Koch-Institut. Händehygiene in einrichtungen des gesundheitswesens. Bundesgesundheitsblatt—Gesundheitsforschung—Gesundheitsschutz. (2016) 59:1189–220. 10.1007/s00103-016-2416-6 27558147 PMC7079999

[19] Dawson-Bowling S Smith J Butt D Cottam H Umasankar S Armitage A. Should outer surgical gloves be changed intraoperatively before orthopaedic prosthesis implantation. J Hosp Infect. (2011) 78:156–7. 10.1016/j.jhin.2011.02.014 21497947

[20] Beldame J Lagrave B Lievain L Lefebvre B Frebourg N Dujardin F. Surgical glove bacterial contamination and perforation during total hip arthroplasty implantation: when gloves should be changed. Orthop Traumatol Surg Res. (2012) 98:432–40. 10.1016/j.otsr.2011.10.015 22578871

[21] Rehman A Rehman AU Rehman TU Freeman C. Removing outer gloves as a method to reduce spinal surgery infection. J Spinal Disord Tech. (2015) 28:E343–6. 10.1097/BSD.0b013e31829046ca 23563341

[22] Anderson PA Savage JW Vaccaro AR Radcliff K Arnold PM Lawrence BD Prevention of surgical site infection in spine surgery. Neurosurgery. (2017) 80:S114–23. 10.1093/neuros/nyw066 28350942

[23] Agarwal A Schultz C Goel VK Agarwal A Anand N Garfin SR Implant prophylaxis: the next best practice toward asepsis in spine surgery. Global Spine J. (2018) 8:761–5. 10.1177/2192568218762380 30443488 PMC6232723

[24] Davis N Curry A Gambhir AK Panigrahi H Walker CR Wilkins EG Intraoperative bacterial contamination in operations for joint replacement. J Bone Joint Surg Br. (1999) 81:886–9. 10.1302/0301-620x.81b5.9545 10530856

[25] Schweitzer D Klaber I Fischman D Wozniak A Botello E Amenábar PP. Surgical light handles: a source of contamination in the surgical field. Acta Orthop Traumatol Turc. (2015) 49:421–5. 10.3944/AOTT.2015.14.0401 26312471

[26] Biswas D Bible JE Whang PG Simpson AK Grauer JN. Sterility of C-arm fluoroscopy during spinal surgery. Spine (Phila Pa 1976). (2008) 33:1913–7. 10.1097/BRS.0b013e31817bb130 18622356

[27] Maria S Deyanira C Francesca S Lucia M Alessandro R Silvia T Spinal fusion surgery and local antibiotic administration: a systematic review on key points from preclinical and clinical data. Spine (Phila Pa 1976). (2020) 45:339–48. 10.1097/BRS.0000000000003255 31568186

[28] Eder C Schenk S Trifinopoulos J Külekci B Kienzl M Schildböck S Does intrawound application of vancomycin influence bone healing in spinal surgery. Eur Spine J. (2016) 25:1021–8. 10.1007/s00586-015-3943-9 25904413

[29] Gande A Rosinski A Cunningham T Bhatia N Lee YP. Selection pressures of vancomycin powder use in spine surgery: a meta-analysis. Spine J. (2019) 19:1076–84. 10.1016/j.spinee.2019.01.002 30660741

[30] Lüdemann M Munoz P Wagner M Malzahn U Horas K Heuschmann P The effect of antiseptics in the prophylaxis of infection in orthopaedic surgery. Z Orthop Unfall. (2018) 156:567–73. 10.1055/a-0608-5292 29902831

[31] Edmiston CE Spencer M Leaper D. Antiseptic irrigation as an effective interventional strategy for reducing the risk of surgical site infections. Surg Infect (Larchmt). (2018) 19:774–80. 10.1089/sur.2018.156 30300563

[32] Lin L Cheng S Wang Y Chen X Zhao G Wang Z Efficacy of intrawound treatments to prevent surgical site infection after spine surgery: a systematic review and network meta-analysis. Pain Physician. (2021) 24:E709–20. 10.36076/ppj.2021.24.E709 34554687

[33] Lemans JVC Wijdicks SPJ Boot W Govaert GAM Houwert RM Öner FC Intrawound treatment for prevention of surgical site infections in instrumented spinal surgery: a systematic comparative effectiveness review and meta-analysis. Global Spine J. (2019) 9:219–30. 10.1177/2192568218786252 30984503 PMC6448203

[34] Onishi Y Masuda K Tozawa K Karita T. Outcomes of an intraoperative povidone-iodine irrigation protocol in spinal surgery for surgical site infection prevention. Clin Spine Surg. (2019) 32:E449–52. 10.1097/BSD.0000000000000908 31609802

[35] Kramer A Dissemond J Kim S Willy C Mayer D Papke R Consensus on wound antisepsis: update 2018. Skin Pharmacol Physiol. (2018) 31:28–58. 10.1159/000481545 29262416

[36] Kramer A. Wundantiseptik: Evidenz, Indikationen, Wirkstoffauswahl und Perspektiven. Ars Med. (2016) 9:419–28.

[37] Wang L Bassiri M Najafi R Najafi K Yang J Khosrovi B Hypochlorous acid as a potential wound care agent: part I. Stabilized hypochlorous acid: a component of the inorganic armamentarium of innate immunity. J Burns Wounds. (2007) 6:e5. 17492050 PMC1853323

[38] Coscia MF Denys GA Wack MF. Propionibacterium acnes, coagulase-negative Staphylococcus, and the “biofilm-like” intervertebral disc. Spine (Phila Pa 1976). (2016) 41:1860–5. 10.1097/BRS.0000000000001909 27669046 PMC5158091

[39] Capoor MN Ruzicka F Schmitz JE James GA Machackova T Jancalek R Propionibacterium acnes biofilm is present in intervertebral discs of patients undergoing microdiscectomy. PLoS One. (2017) 12:e0174518. 10.1371/journal.pone.0174518 28369127 PMC5378350

[40] Walters R Moore R Fraser R. Penetration of cephazolin in human lumbar intervertebral disc. Spine. (2006) 31:567–70. 10.1097/01.brs.0000201244.24003.2d 16508553

[41] Capoor MN Lochman J McDowell A Schmitz JE Solansky M Zapletalova M Intervertebral disc penetration by antibiotics used prophylactically in spinal surgery: implications for the current standards and treatment of disc infections. Eur Spine J. (2019) 28:783–91. 10.1007/s00586-018-5838-z 30506486

[42] Nakase K Fukushima H Yukawa T Nakaminami H Fujii T Noguchi N. Propionibacterium acnes has low susceptibility to chlorhexidine digluconate. Surg Infect (Larchmt). (2018) 19:298–302. 10.1089/sur.2017.220 29447075

[43] Casey AL Karpanen TJ Nightingale P Conway BR Elliott TS. Antimicrobial activity and skin permeation of iodine present in an iodine-impregnated surgical incise drape. J Antimicrob Chemother. (2015) 70:2255–60. 10.1093/jac/dkv100 25904727

[44] Hesselvig AB Arpi M Madsen F Bjarnsholt T Odgaard A, ICON SG. Does an antimicrobial incision drape prevent intraoperative contamination? A randomized controlled trial of 1187 patients. Clin Orthop Relat Res. (2020) 478(5):1007–15. 10.1097/CORR.0000000000001142 32011378 PMC7170680

[45] Rezapoor M Tan TL Maltenfort MG Parvizi J. Incise draping reduces the rate of contamination of the surgical site during hip surgery: a prospective, randomized trial. J Arthroplasty. (2018) 33:1891–5. 10.1016/j.arth.2018.01.013 29525345

[46] Agarwal A Lin B Wang JC Schultz C Garfin SR Goel VK Efficacy of intraoperative implant prophylaxis in reducing intraoperative microbial contamination. Global Spine J. (2019) 9:62–6. 10.1177/2192568218780676 30775210 PMC6362554

[47] Dalstrom DJ Venkatarayappa I Manternach AL Palcic MS Heyse BA Prayson MJ. Time-dependent contamination of opened sterile operating-room trays. J Bone Joint Surg Am. (2008) 90:1022–5. 10.2106/JBJS.G.00689 18451394

[48] Bible JE O’Neill KR Crosby CG Schoenecker JG McGirt MJ Devin CJ. Implant contamination during spine surgery. Spine J. (2013) 13:637–40. 10.1016/j.spinee.2012.11.053 23321148

[49] Menekse G Kuscu F Suntur BM Gezercan Y Ates T Ozsoy KM Evaluation of the time-dependent contamination of spinal implants: prospective randomized trial. Spine (Phila Pa 1976). (2015) 40:1247–51. 10.1097/BRS.0000000000000944 25929209

[50] Brown AR Taylor GJ Gregg PJ. Air contamination during skin preparation and draping in joint replacement surgery. J Bone Joint Surg Br. (1996) 78:92–4. 10.1302/0301-620X.78B1.0780092 8898135

[51] Ward JP Feldman DS Paul J Sala DA Errico TJ Otsuka NY Wound closure in nonidiopathic scoliosis: does closure matter? J Pediatr Orthop. (2015) 37(3):166–70. 10.1097/BPO.0000000000000610 26214326

[52] Rao SB Vasquez G Harrop J Maltenfort M Stein N Kaliyadan G Risk factors for surgical site infections following spinal fusion procedures: a case-control study. Clin Infect Dis. (2011) 53:686–92. 10.1093/cid/cir506 21890772

[53] Liu JM Deng HL Chen XY Zhou Y Yang D Duan MS Risk factors for surgical site infection after posterior lumbar spinal surgery. Spine (Phila Pa 1976). (2018) 43:732–7. 10.1097/BRS.0000000000002419 28922276

[54] Tan T Lee H Huang MS Rutges J Marion TE Mathew J Prophylactic postoperative measures to minimize surgical site infections in spine surgery: systematic review and evidence summary. Spine J. (2019). 20(3):435–47. 10.1016/j.spinee.2019.09.013 31557586

[55] Adogwa O Fatemi P Perez E Moreno J Gazcon GC Gokaslan ZL Negative pressure wound therapy reduces incidence of postoperative wound infection and dehiscence after long-segment thoracolumbar spinal fusion: a single institutional experience. Spine J. (2014) 14:2911–7. 10.1016/j.spinee.2014.04.011 24769401

[56] Naylor RM Gilder HE Gupta N Hydrick TC Labott JR Mauler DJ Effects of negative pressure wound therapy on wound dehiscence and surgical site infection following instrumented spinal fusion surgery—a single surgeon’s experience. World Neurosurg. (2020) 137:e257–62. 10.1016/j.wneu.2020.01.152 32004742 PMC8063507

[57] Dyck BA Bailey CS Steyn C Petrakis J Urquhart JC Raj R Use of incisional vacuum-assisted closure in the prevention of postoperative infection in high-risk patients who underwent spine surgery: a proof-of-concept study. J Neurosurg Spine. (2019) 31:430–9. 10.3171/2019.2.SPINE18947 31075767

[58] Stannard JP Gabriel A Lehner B. Use of negative pressure wound therapy over clean, closed surgical incisions. Int Wound J. (2012) 9(Suppl 1):32–9. 10.1111/j.1742-481X.2012.01017.x 22727138 PMC7950357

